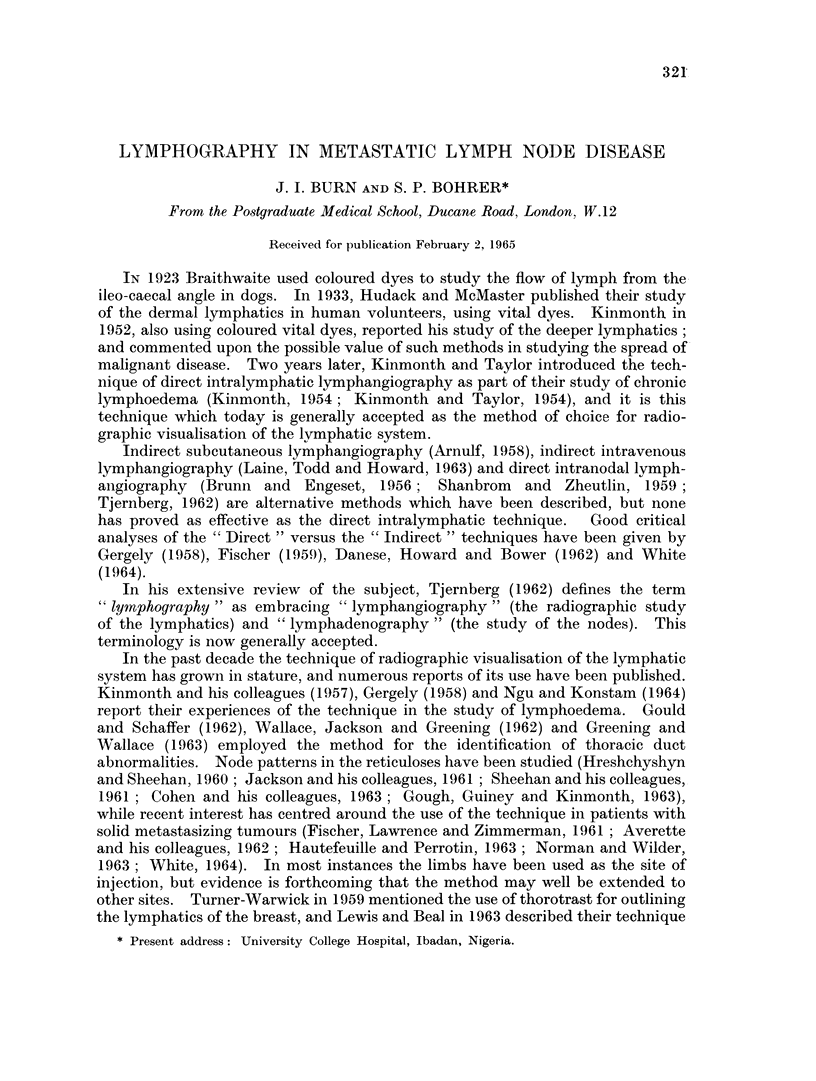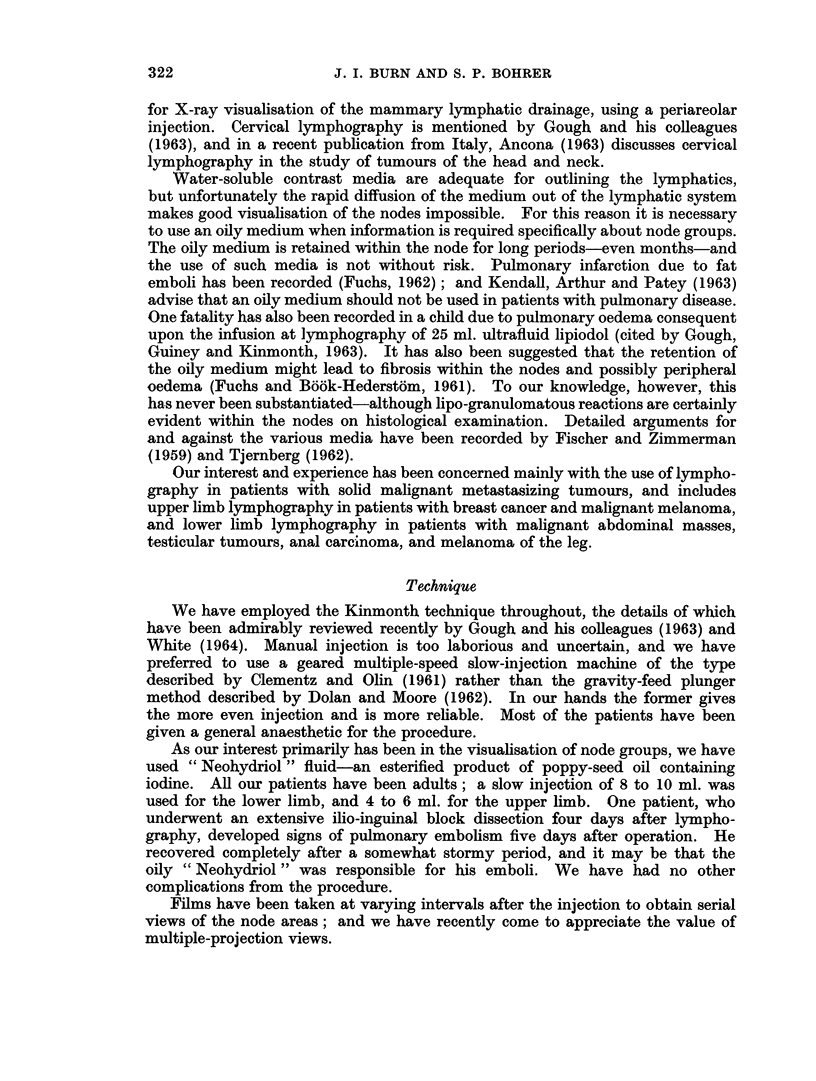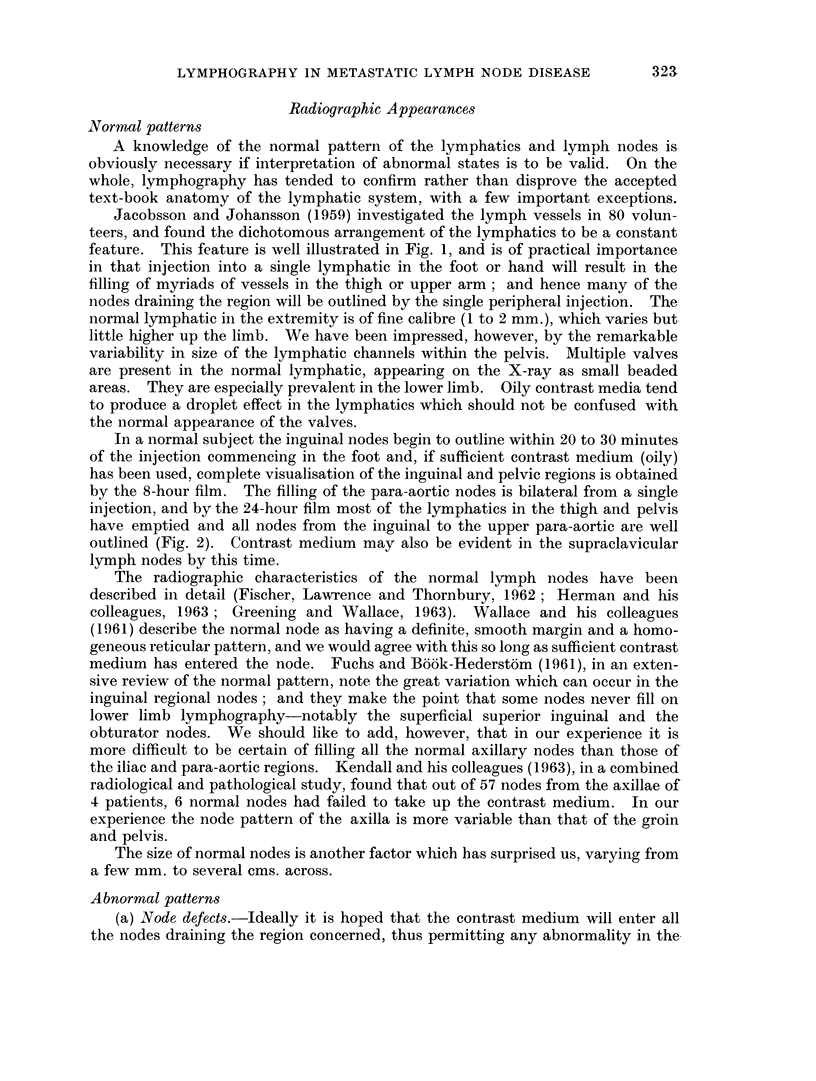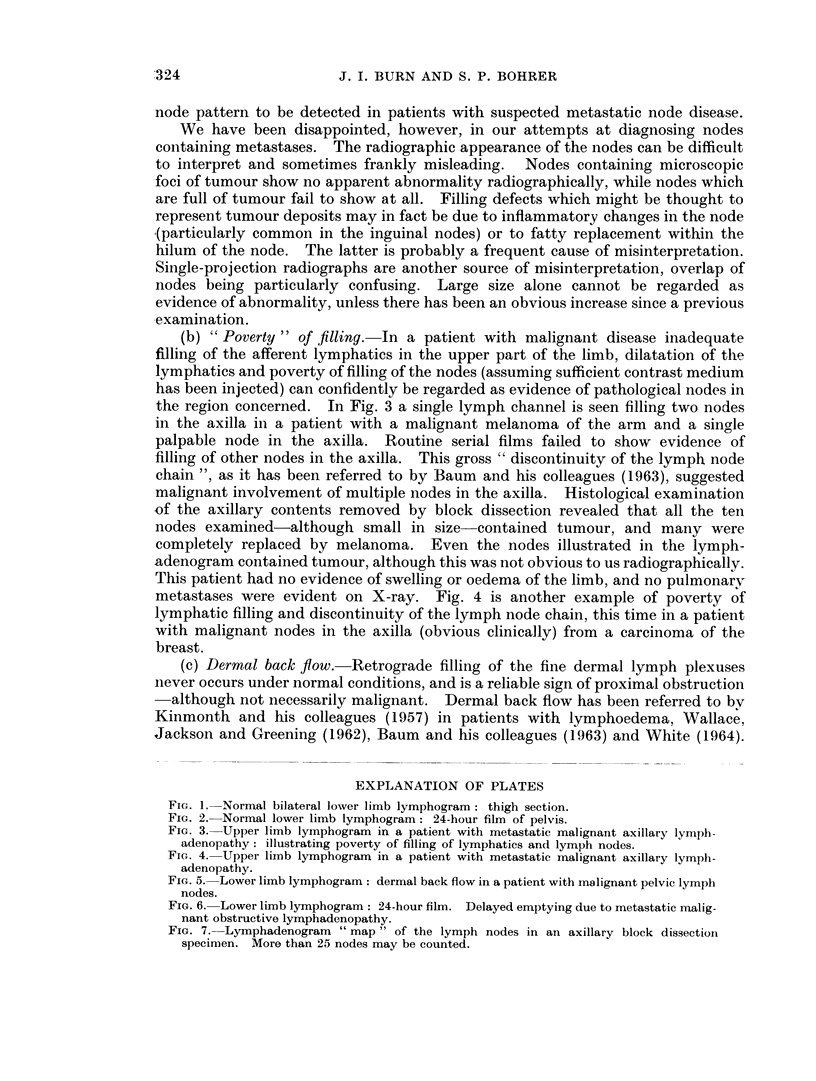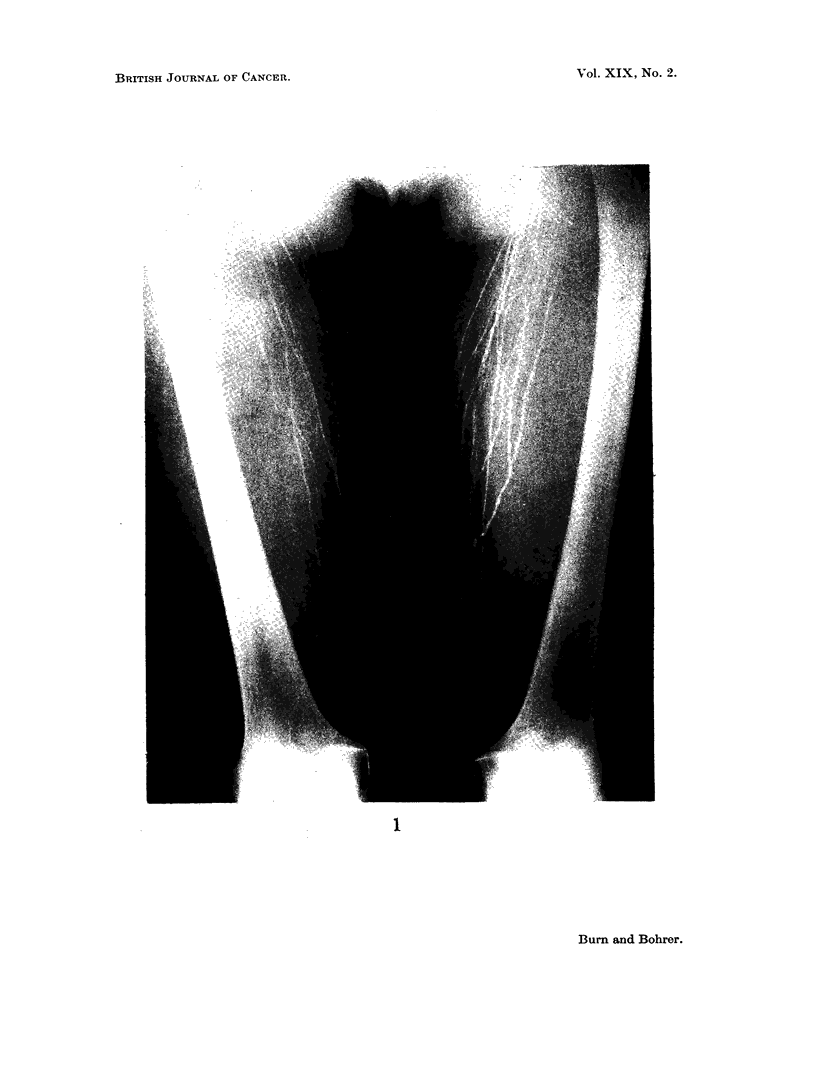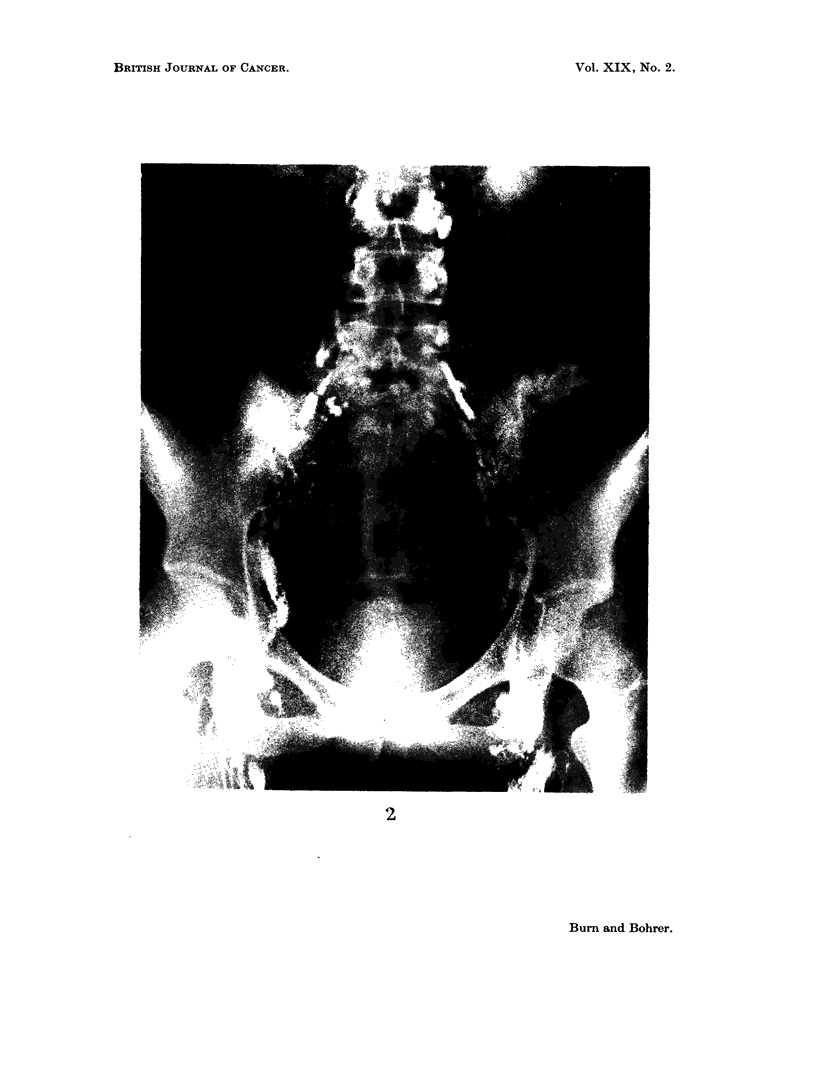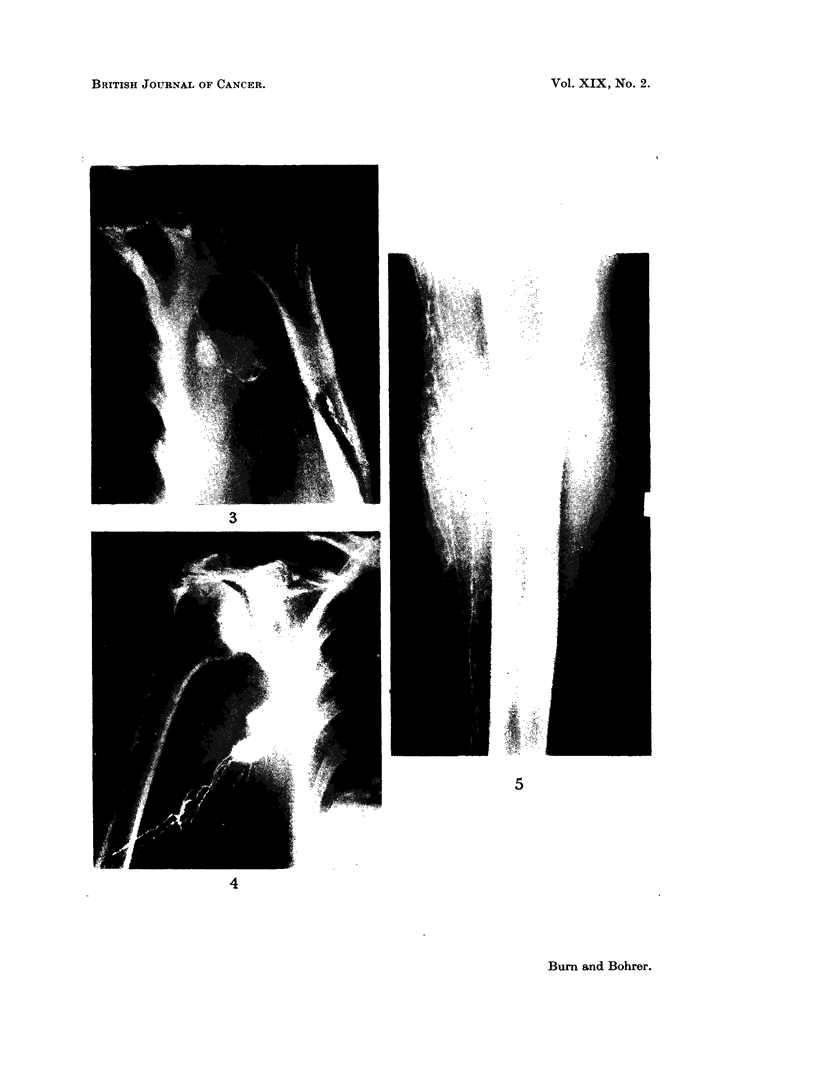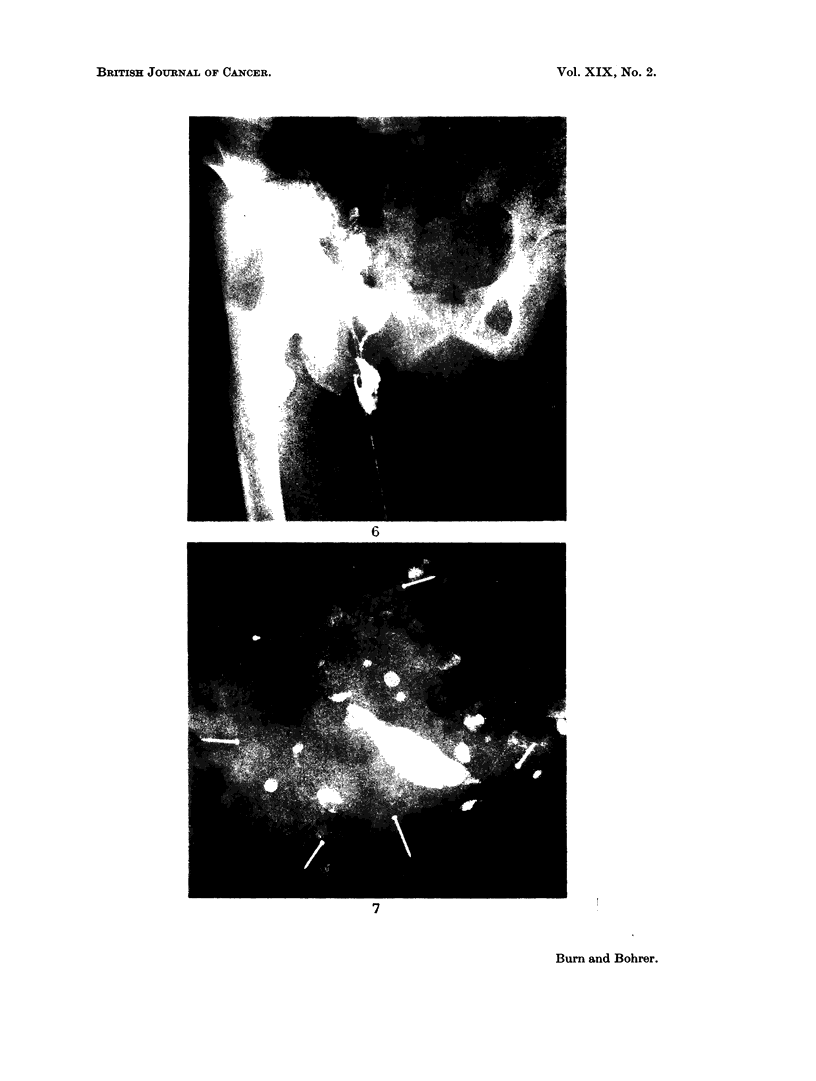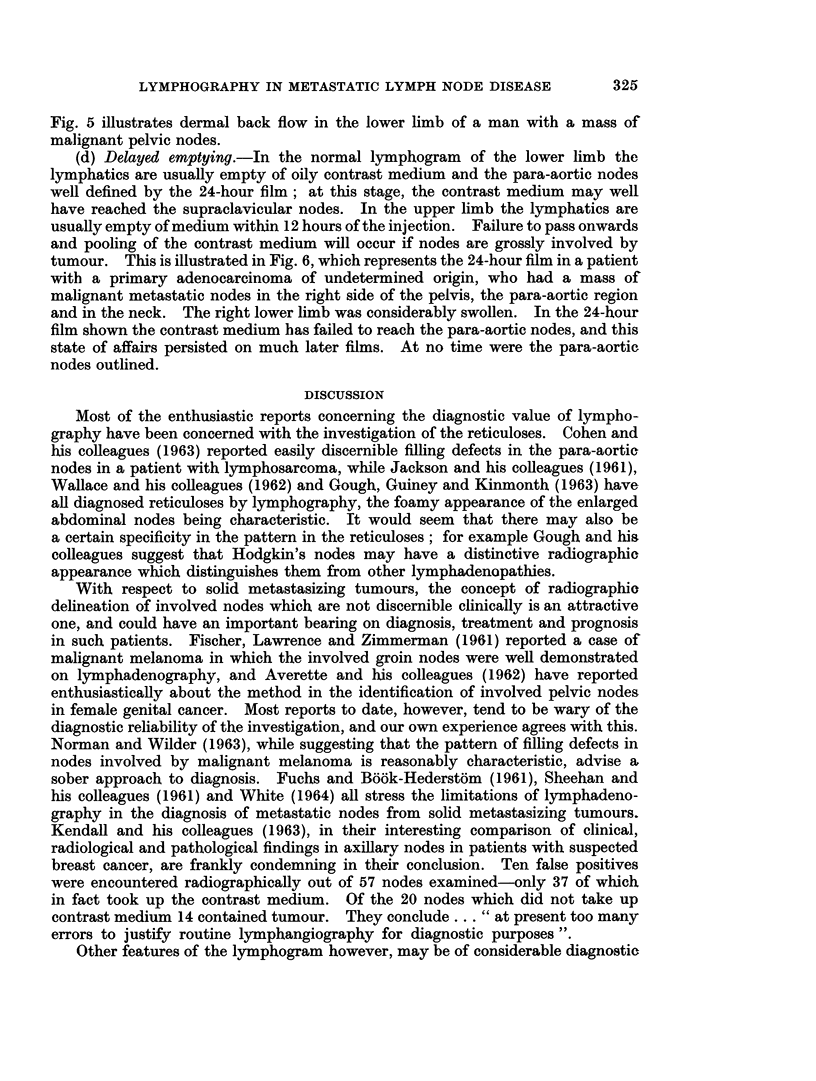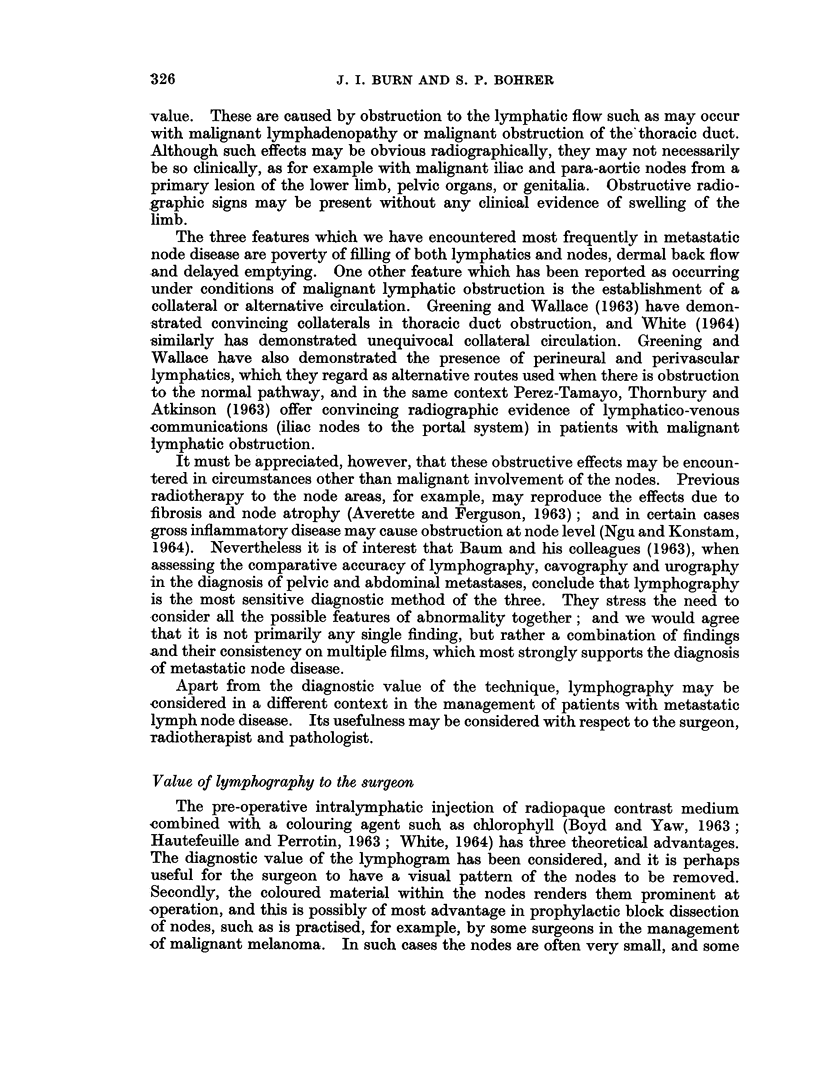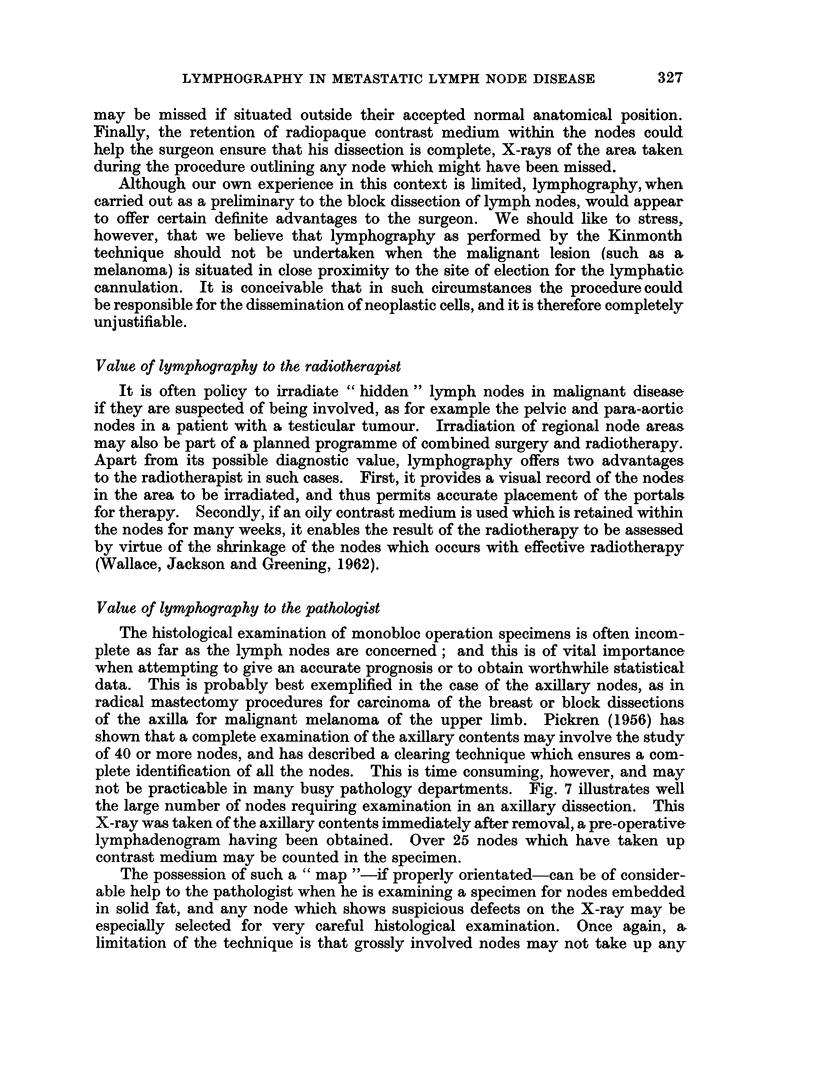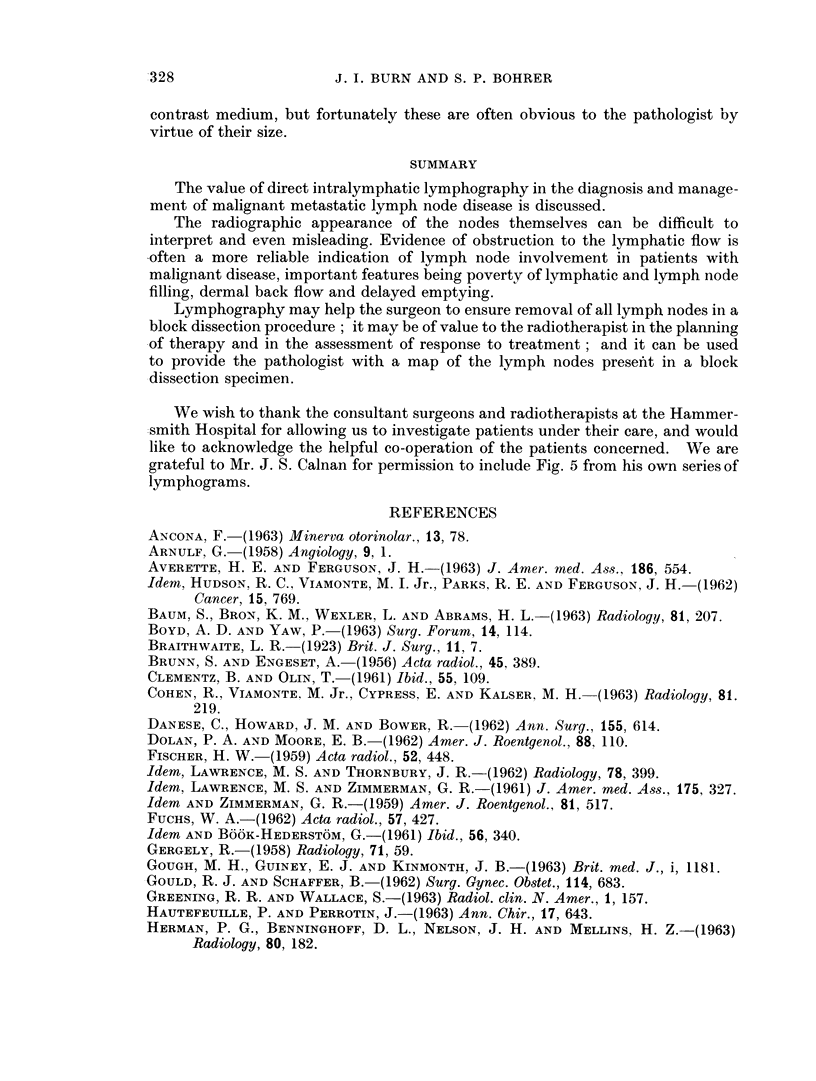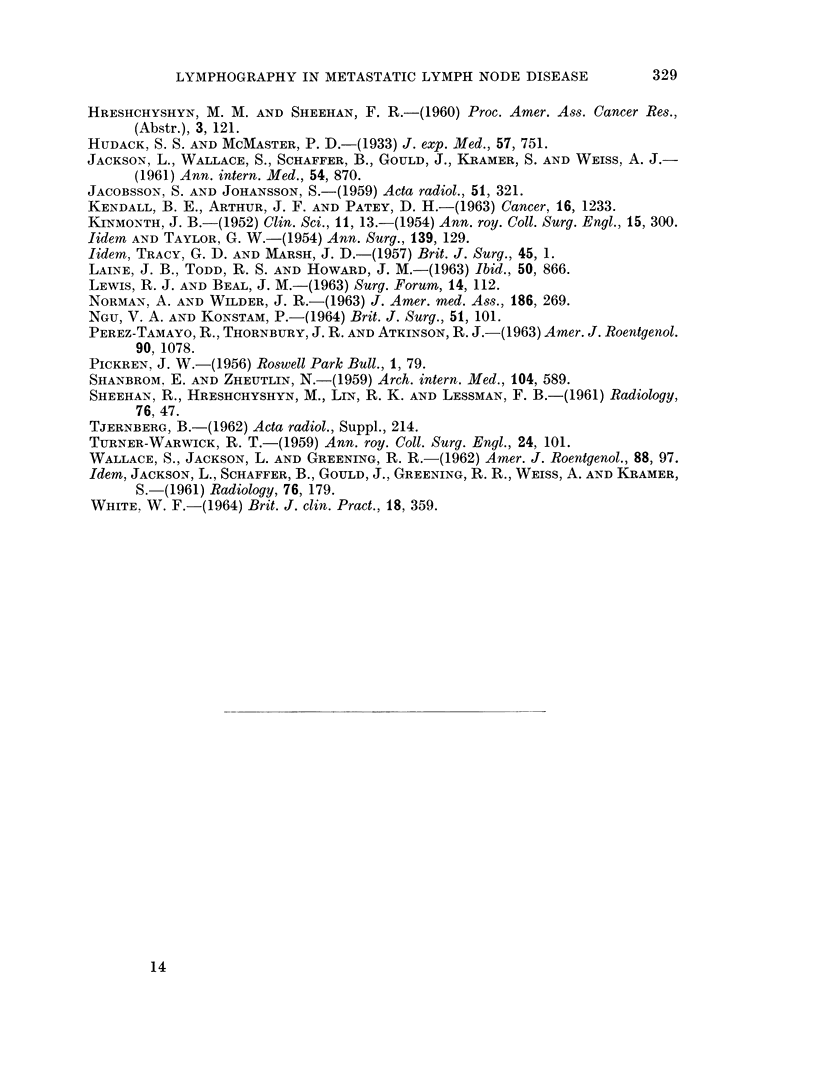# Lymphography in Metastatic Lymph Node Disease

**DOI:** 10.1038/bjc.1965.37

**Published:** 1965-06

**Authors:** J. I. Burn, S. P. Bohrer

## Abstract

**Images:**


### LYMPHOGRAPHY IN METASTATIC LYMPH NODE DISEASE

J. I. BURN AND S. P. BOHRER*

*From the Postgraduate Medical School, Ducane Road, London, W.12*

Received for publication February 2, 1965

IN 1923 Braithwaite used coloured dyes to study the flow of lymph from the ileo-caecal angle in dogs. In 1933, Hudack and McMaster published their study of the dermal lymphatics in human volunteers, using vital dyes. Kinmonth in 1952, also using coloured vital dyes, reported his study of the deeper lymphatics ; and commented upon the possible value of such methods in studying the spread of malignant disease. Two years later, Kinmonth and Taylor introduced the technique of direct intralymphatic lymphangiography as part of their study of chronic lymphoedema (Kinmonth, 1954 ; Kinmonth and Taylor, 1954), and it is this technique which today is generally accepted as the method of choice for radiographic visualisation of the lymphatic system.

Indirect subcutaneous lymphangiography (Arnulf, 1958), indirect intravenous lymphangiography (Laine, Todd and Howard, 1963) and direct intranodal lymphangiography (Brunn and Engeset, 1956 ; Shanbrom and Zheutlin, 1959 ; Tjernberg, 1962) are alternative methods which have been described, but none has proved as effective as the direct intralymphatic technique. Good critical analyses of the " Direct " versus the " Indirect " techniques have been given by Gergely (1958), Fischer (1959), Danese, Howard and Bower (1962) and White (1964).

In his extensive review of the subject, Tjernberg (1962) defines the term " *lymphography* " as embracing " lymphangiography " (the radiographic study of the lymphatics) and " lymphadenography " (the study of the nodes). This terminology is now generally accepted.

In the past decade the technique of radiographic visualisation of the lymphatic system has grown in stature, and numerous reports of its use have been published. Kinmonth and his colleagues (1957), Gergely (1958) and Ngu and Konstam (1964) report their experiences of the technique in the study of lymphoedema. Gould and Schaffer (1962), Wallace, Jackson and Greening (1962) and Greening and Wallace (1963) employed the method for the identification of thoracic duct abnormalities. Node patterns in the reticuloses have been studied (Hreshchyshyn and Sheehan, 1960 ; Jackson and his colleagues, 1961 ; Sheehan and his colleagues, 1961 ; Cohen and his colleagues, 1963 ; Gough, Guiney and Kinmonth, 1963), while recent interest has centred around the use of the technique in patients with solid metastasizing tumours (Fischer, Lawrence and Zimmerman, 1961 ; Averette and his colleagues, 1962 ; Hautefeuille and Perrotin, 1963 ; Norman and Wilder, 1963 ; White, 1964). In most instances the limbs have been used as the site of injection, but evidence is forthcoming that the method may well be extended to other sites. Turner-Warwick in 1959 mentioned the use of thorotrast for outlining the lymphatics of the breast, and Lewis and Beal in 1963 described their technique

* Present address : University College Hospital, Ibadan, Nigeria.

for X-ray visualisation of the mammary lymphatic drainage, using a periareolar injection. Cervical lymphography is mentioned by Gough and his colleagues (1963), and in a recent publication from Italy, Ancona (1963) discusses cervical lymphography in the study of tumours of the head and neck.

Water-soluble contrast media are adequate for outlining the lymphatics, but unfortunately the rapid diffusion of the medium out of the lymphatic system makes good visualisation of the nodes impossible. For this reason it is necessary to use an oily medium when information is required specifically about node groups. The oily medium is retained within the node for long periods—even months—and the use of such media is not without risk. Pulmonary infarction due to fat emboli has been recorded (Fuchs, 1962) ; and Kendall, Arthur and Patey (1963) advise that an oily medium should not be used in patients with pulmonary disease. One fatality has also been recorded in a child due to pulmonary oedema consequent upon the infusion at lymphography of 25 ml. ultrafluid lipiodol (cited by Gough, Guiney and Kinmonth, 1963). It has also been suggested that the retention of the oily medium might lead to fibrosis within the nodes and possibly peripheral oedema (Fuchs and Böök-Hederstöm, 1961). To our knowledge, however, this has never been substantiated—although lipo-granulomatous reactions are certainly evident within the nodes on histological examination. Detailed arguments for and against the various media have been recorded by Fischer and Zimmerman (1959) and Tjernberg (1962).

Our interest and experience has been concerned mainly with the use of lymphography in patients with solid malignant metastasizing tumours, and includes upper limb lymphography in patients with breast cancer and malignant melanoma, and lower limb lymphography in patients with malignant abdominal masses, testicular tumours, anal carcinoma, and melanoma of the leg.

### *Technique*

We have employed the Kinmonth technique throughout, the details of which have been admirably reviewed recently by Gough and his colleagues (1963) and White (1964). Manual injection is too laborious and uncertain, and we have preferred to use a geared multiple-speed slow-injection machine of the type described by Clementz and Olin (1961) rather than the gravity-feed plunger method described by Dolan and Moore (1962). In our hands the former gives the more even injection and is more reliable. Most of the patients have been given a general anaesthetic for the procedure.

As our interest primarily has been in the visualisation of node groups, we have used " Neohydriol " fluid—an esterified product of poppy-seed oil containing iodine. All our patients have been adults ; a slow injection of 8 to 10 ml. was used for the lower limb, and 4 to 6 ml. for the upper limb. One patient, who underwent an extensive ilio-inguinal block dissection four days after lymphography, developed signs of pulmonary embolism five days after operation. He recovered completely after a somewhat stormy period, and it may be that the oily " Neohydriol " was responsible for his emboli. We have had no other complications from the procedure.

Films have been taken at varying intervals after the injection to obtain serial views of the node areas ; and we have recently come to appreciate the value of multiple-projection views.

### *Radiographic Appearances*

### *Normal patterns*

A knowledge of the normal pattern of the lymphatics and lymph nodes is obviously necessary if interpretation of abnormal states is to be valid. On the whole, lymphography has tended to confirm rather than disprove the accepted text-book anatomy of the lymphatic system, with a few important exceptions.

Jacobsson and Johansson (1959) investigated the lymph vessels in 80 volunteers, and found the dichotomous arrangement of the lymphatics to be a constant feature. This feature is well illustrated in Fig. 1, and is of practical importance in that injection into a single lymphatic in the foot or hand will result in the filling of myriads of vessels in the thigh or upper arm ; and hence many of the nodes draining the region will be outlined by the single peripheral injection. The normal lymphatic in the extremity is of fine calibre (1 to 2 mm.), which varies but little higher up the limb. We have been impressed, however, by the remarkable variability in size of the lymphatic channels within the pelvis. Multiple valves are present in the normal lymphatic, appearing on the X-ray as small beaded areas. They are especially prevalent in the lower limb. Oily contrast media tend to produce a droplet effect in the lymphatics which should not be confused with the normal appearance of the valves.

In a normal subject the inguinal nodes begin to outline within 20 to 30 minutes of the injection commencing in the foot and, if sufficient contrast medium (oily) has been used, complete visualisation of the inguinal and pelvic regions is obtained by the 8-hour film. The filling of the para-aortic nodes is bilateral from a single injection, and by the 24-hour film most of the lymphatics in the thigh and pelvis have emptied and all nodes from the inguinal to the upper para-aortic are well outlined (Fig. 2). Contrast medium may also be evident in the supraclavicular lymph nodes by this time.

The radiographic characteristics of the normal lymph nodes have been described in detail (Fischer, Lawrence and Thornbury, 1962 ; Herman and his colleagues, 1963 ; Greening and Wallace, 1963). Wallace and his colleagues (1961) describe the normal node as having a definite, smooth margin and a homogeneous reticular pattern, and we would agree with this so long as sufficient contrast medium has entered the node. Fuchs and Böök-Hederstöm (1961), in an extensive review of the normal pattern, note the great variation which can occur in the inguinal regional nodes ; and they make the point that some nodes never fill on lower limb lymphography—notably the superficial superior inguinal and the obturator nodes. We should like to add, however, that in our experience it is more difficult to be certain of filling all the normal axillary nodes than those of the iliac and para-aortic regions. Kendall and his colleagues (1963), in a combined radiological and pathological study, found that out of 57 nodes from the axillae of 4 patients, 6 normal nodes had failed to take up the contrast medium. In our experience the node pattern of the axilla is more variable than that of the groin and pelvis.

The size of normal nodes is another factor which has surprised us, varying from a few mm. to several cms. across.

### *Abnormal patterns*

(a) *Node defects.*—Ideally it is hoped that the contrast medium will enter all the nodes draining the region concerned, thus permitting any abnormality in the node pattern to be detected in patients with suspected metastatic node disease.

We have been disappointed, however, in our attempts at diagnosing nodes containing metastases. The radiographic appearance of the nodes can be difficult to interpret and sometimes frankly misleading. Nodes containing microscopic foci of tumour show no apparent abnormality radiographically, while nodes which are full of tumour fail to show at all. Filling defects which might be thought to represent tumour deposits may in fact be due to inflammatory changes in the node (particularly common in the inguinal nodes) or to fatty replacement within the hilum of the node. The latter is probably a frequent cause of misinterpretation. Single-projection radiographs are another source of misinterpretation, overlap of nodes being particularly confusing. Large size alone cannot be regarded as evidence of abnormality, unless there has been an obvious increase since a previous examination.

(b) *"Poverty" of filling.*—In a patient with malignant disease inadequate filling of the afferent lymphatics in the upper part of the limb, dilatation of the lymphatics and poverty of filling of the nodes (assuming sufficient contrast medium has been injected) can confidently be regarded as evidence of pathological nodes in the region concerned. In Fig. 3 a single lymph channel is seen filling two nodes in the axilla in a patient with a malignant melanoma of the arm and a single palpable node in the axilla. Routine serial films failed to show evidence of filling of other nodes in the axilla. This gross "discontinuity of the lymph node chain", as it has been referred to by Baum and his colleagues (1963), suggested malignant involvement of multiple nodes in the axilla. Histological examination of the axillary contents removed by block dissection revealed that all the ten nodes examined—although small in size—contained tumour, and many were completely replaced by melanoma. Even the nodes illustrated in the lymphadenogram contained tumour, although this was not obvious to us radiographically. This patient had no evidence of swelling or oedema of the limb, and no pulmonary metastases were evident on X-ray. Fig. 4 is another example of poverty of lymphatic filling and discontinuity of the lymph node chain, this time in a patient with malignant nodes in the axilla (obvious clinically) from a carcinoma of the breast.

(c) *Dermal back flow.*—Retrograde filling of the fine dermal lymph plexuses never occurs under normal conditions, and is a reliable sign of proximal obstruction —although not necessarily malignant. Dermal back flow has been referred to by Kinmonth and his colleagues (1957) in patients with lymphoedema, Wallace, Jackson and Greening (1962), Baum and his colleagues (1963) and White (1964).

---

EXPLANATION OF PLATES

FIG. 1.—Normal bilateral lower limb lymphogram: thigh section.

FIG. 2.—Normal lower limb lymphogram: 24-hour film of pelvis.

FIG. 3.—Upper limb lymphogram in a patient with metastatic malignant axillary lymphadenopathy: illustrating poverty of filling of lymphatics and lymph nodes.

FIG. 4.—Upper limb lymphogram in a patient with metastatic malignant axillary lymphadenopathy.

FIG. 5.—Lower limb lymphogram: dermal back flow in a patient with malignant pelvic lymph nodes.

FIG. 6.—Lower limb lymphogram: 24-hour film. Delayed emptying due to metastatic malignant obstructive lymphadenopathy.

FIG. 7.—Lymphadenogram "map" of the lymph nodes in an axillary block dissection specimen. More than 25 nodes may be counted.

1

Burn and Bohrer.

2

Burn and Bohrer.

3

4

5

Burn and Bohrer.

6

7

Fig. 5 illustrates dermal back flow in the lower limb of a man with a mass of malignant pelvic nodes.

(d) *Delayed emptying*.—In the normal lymphogram of the lower limb the lymphatics are usually empty of oily contrast medium and the para-aortic nodes well defined by the 24-hour film ; at this stage, the contrast medium may well have reached the supraclavicular nodes. In the upper limb the lymphatics are usually empty of medium within 12 hours of the injection. Failure to pass onwards and pooling of the contrast medium will occur if nodes are grossly involved by tumour. This is illustrated in Fig. 6, which represents the 24-hour film in a patient with a primary adenocarcinoma of undetermined origin, who had a mass of malignant metastatic nodes in the right side of the pelvis, the para-aortic region and in the neck. The right lower limb was considerably swollen. In the 24-hour film shown the contrast medium has failed to reach the para-aortic nodes, and this state of affairs persisted on much later films. At no time were the para-aortic nodes outlined.

### DISCUSSION

Most of the enthusiastic reports concerning the diagnostic value of lymphography have been concerned with the investigation of the reticuloses. Cohen and his colleagues (1963) reported easily discernible filling defects in the para-aortic nodes in a patient with lymphosarcoma, while Jackson and his colleagues (1961), Wallace and his colleagues (1962) and Gough, Guiney and Kinmonth (1963) have all diagnosed reticuloses by lymphography, the foamy appearance of the enlarged abdominal nodes being characteristic. It would seem that there may also be a certain specificity in the pattern in the reticuloses ; for example Gough and his colleagues suggest that Hodgkin's nodes may have a distinctive radiographic appearance which distinguishes them from other lymphadenopathies.

With respect to solid metastasizing tumours, the concept of radiographic delineation of involved nodes which are not discernible clinically is an attractive one, and could have an important bearing on diagnosis, treatment and prognosis in such patients. Fischer, Lawrence and Zimmerman (1961) reported a case of malignant melanoma in which the involved groin nodes were well demonstrated on lymphadenography, and Averette and his colleagues (1962) have reported enthusiastically about the method in the identification of involved pelvic nodes in female genital cancer. Most reports to date, however, tend to be wary of the diagnostic reliability of the investigation, and our own experience agrees with this. Norman and Wilder (1963), while suggesting that the pattern of filling defects in nodes involved by malignant melanoma is reasonably characteristic, advise a sober approach to diagnosis. Fuchs and Böök-Hederstöm (1961), Sheehan and his colleagues (1961) and White (1964) all stress the limitations of lymphadenography in the diagnosis of metastatic nodes from solid metastasizing tumours. Kendall and his colleagues (1963), in their interesting comparison of clinical, radiological and pathological findings in axillary nodes in patients with suspected breast cancer, are frankly condemning in their conclusion. Ten false positives were encountered radiographically out of 57 nodes examined—only 37 of which in fact took up the contrast medium. Of the 20 nodes which did not take up contrast medium 14 contained tumour. They conclude . . . " at present too many errors to justify routine lymphangiography for diagnostic purposes ".

Other features of the lymphogram however, may be of considerable diagnostic value. These are caused by obstruction to the lymphatic flow such as may occur with malignant lymphadenopathy or malignant obstruction of the thoracic duct. Although such effects may be obvious radiographically, they may not necessarily be so clinically, as for example with malignant iliac and para-aortic nodes from a primary lesion of the lower limb, pelvic organs, or genitalia. Obstructive radiographic signs may be present without any clinical evidence of swelling of the limb.

The three features which we have encountered most frequently in metastatic node disease are poverty of filling of both lymphatics and nodes, dermal back flow and delayed emptying. One other feature which has been reported as occurring under conditions of malignant lymphatic obstruction is the establishment of a collateral or alternative circulation. Greening and Wallace (1963) have demonstrated convincing collaterals in thoracic duct obstruction, and White (1964) similarly has demonstrated unequivocal collateral circulation. Greening and Wallace have also demonstrated the presence of perineural and perivascular lymphatics, which they regard as alternative routes used when there is obstruction to the normal pathway, and in the same context Perez-Tamayo, Thornbury and Atkinson (1963) offer convincing radiographic evidence of lymphatico-venous communications (iliac nodes to the portal system) in patients with malignant lymphatic obstruction.

It must be appreciated, however, that these obstructive effects may be encountered in circumstances other than malignant involvement of the nodes. Previous radiotherapy to the node areas, for example, may reproduce the effects due to fibrosis and node atrophy (Averette and Ferguson, 1963) ; and in certain cases gross inflammatory disease may cause obstruction at node level (Ngu and Konstam, 1964). Nevertheless it is of interest that Baum and his colleagues (1963), when assessing the comparative accuracy of lymphography, cavography and urography in the diagnosis of pelvic and abdominal metastases, conclude that lymphography is the most sensitive diagnostic method of the three. They stress the need to consider all the possible features of abnormality together ; and we would agree that it is not primarily any single finding, but rather a combination of findings and their consistency on multiple films, which most strongly supports the diagnosis of metastatic node disease.

Apart from the diagnostic value of the technique, lymphography may be considered in a different context in the management of patients with metastatic lymph node disease. Its usefulness may be considered with respect to the surgeon, radiotherapist and pathologist.

*Value of lymphography to the surgeon*

The pre-operative intralymphatic injection of radiopaque contrast medium combined with a colouring agent such as chlorophyll (Boyd and Yaw, 1963 ; Hautefeuille and Perrotin, 1963 ; White, 1964) has three theoretical advantages. The diagnostic value of the lymphogram has been considered, and it is perhaps useful for the surgeon to have a visual pattern of the nodes to be removed. Secondly, the coloured material within the nodes renders them prominent at operation, and this is possibly of most advantage in prophylactic block dissection of nodes, such as is practised, for example, by some surgeons in the management of malignant melanoma. In such cases the nodes are often very small, and some may be missed if situated outside their accepted normal anatomical position. Finally, the retention of radiopaque contrast medium within the nodes could help the surgeon ensure that his dissection is complete, X-rays of the area taken during the procedure outlining any node which might have been missed.

Although our own experience in this context is limited, lymphography, when carried out as a preliminary to the block dissection of lymph nodes, would appear to offer certain definite advantages to the surgeon. We should like to stress, however, that we believe that lymphography as performed by the Kinmonth technique should not be undertaken when the malignant lesion (such as a melanoma) is situated in close proximity to the site of election for the lymphatic cannulation. It is conceivable that in such circumstances the procedure could be responsible for the dissemination of neoplastic cells, and it is therefore completely unjustifiable.

*Value of lymphography to the radiotherapist*

It is often policy to irradiate " hidden " lymph nodes in malignant disease if they are suspected of being involved, as for example the pelvic and para-aortic nodes in a patient with a testicular tumour. Irradiation of regional node areas may also be part of a planned programme of combined surgery and radiotherapy. Apart from its possible diagnostic value, lymphography offers two advantages to the radiotherapist in such cases. First, it provides a visual record of the nodes in the area to be irradiated, and thus permits accurate placement of the portals for therapy. Secondly, if an oily contrast medium is used which is retained within the nodes for many weeks, it enables the result of the radiotherapy to be assessed by virtue of the shrinkage of the nodes which occurs with effective radiotherapy (Wallace, Jackson and Greening, 1962).

*Value of lymphography to the pathologist*

The histological examination of monobloc operation specimens is often incomplete as far as the lymph nodes are concerned ; and this is of vital importance when attempting to give an accurate prognosis or to obtain worthwhile statistical data. This is probably best exemplified in the case of the axillary nodes, as in radical mastectomy procedures for carcinoma of the breast or block dissections of the axilla for malignant melanoma of the upper limb. Pickren (1956) has shown that a complete examination of the axillary contents may involve the study of 40 or more nodes, and has described a clearing technique which ensures a complete identification of all the nodes. This is time consuming, however, and may not be practicable in many busy pathology departments. Fig. 7 illustrates well the large number of nodes requiring examination in an axillary dissection. This X-ray was taken of the axillary contents immediately after removal, a pre-operative lymphadenogram having been obtained. Over 25 nodes which have taken up contrast medium may be counted in the specimen.

The possession of such a " map "—if properly orientated—can be of considerable help to the pathologist when he is examining a specimen for nodes embedded in solid fat, and any node which shows suspicious defects on the X-ray may be especially selected for very careful histological examination. Once again, a limitation of the technique is that grossly involved nodes may not take up any contrast medium, but fortunately these are often obvious to the pathologist by virtue of their size.

### SUMMARY

The value of direct intralymphatic lymphography in the diagnosis and management of malignant metastatic lymph node disease is discussed.

The radiographic appearance of the nodes themselves can be difficult to interpret and even misleading. Evidence of obstruction to the lymphatic flow is often a more reliable indication of lymph node involvement in patients with malignant disease, important features being poverty of lymphatic and lymph node filling, dermal back flow and delayed emptying.

Lymphography may help the surgeon to ensure removal of all lymph nodes in a block dissection procedure ; it may be of value to the radiotherapist in the planning of therapy and in the assessment of response to treatment ; and it can be used to provide the pathologist with a map of the lymph nodes present in a block dissection specimen.

We wish to thank the consultant surgeons and radiotherapists at the Hammersmith Hospital for allowing us to investigate patients under their care, and would like to acknowledge the helpful co-operation of the patients concerned. We are grateful to Mr. J. S. Calnan for permission to include Fig. 5 from his own series of lymphograms.

### REFERENCES

Ancona, F.—(1963) *Minerva otorinolar.*, **13**, 78.

Arnulf, G.—(1958) *Angiology*, **9**, 1.

Averette, H. E. and Ferguson, J. H.—(1963) *J. Amer. med. Ass.*, **186**, 554.

*Idem*, Hudson, R. C., Viamonte, M. I. Jr., Parks, R. E. and Ferguson, J. H.—(1962) *Cancer*, **15**, 769.

Baum, S., Bron, K. M., Wexler, L. and Abrams, H. L.—(1963) *Radiology*, **81**, 207.

Boyd, A. D. and Yaw, P.—(1963) *Surg. Forum*, **14**, 114.

Braithwaite, L. R.—(1923) *Brit. J. Surg.*, **11**, 7.

Brunn, S. and Engeset, A.—(1956) *Acta radiol.*, **45**, 389.

Clementz, B. and Olin, T.—(1961) *Ibid.*, **55**, 109.

Cohen, R., Viamonte, M. Jr., Cypress, E. and Kalser, M. H.—(1963) *Radiology*, **81**. 219.

Danese, C., Howard, J. M. and Bower, R.—(1962) *Ann. Surg.*, **155**, 614.

Dolan, P. A. and Moore, E. B.—(1962) *Amer. J. Roentgenol.*, **88**, 110.

Fischer, H. W.—(1959) *Acta radiol.*, **52**, 448.

*Idem*, Lawrence, M. S. and Thornbury, J. R.—(1962) *Radiology*, **78**, 399.

*Idem*, Lawrence, M. S. and Zimmerman, G. R.—(1961) *J. Amer. med. Ass.*, **175**, 327.

*Idem* and Zimmerman, G. R.—(1959) *Amer. J. Roentgenol.*, **81**, 517.

Fuchs, W. A.—(1962) *Acta radiol.*, **57**, 427.

*Idem* and Böök-Hederstöm, G.—(1961) *Ibid.*, **56**, 340.

Gergely, R.—(1958) *Radiology*, **71**, 59.

Gough, M. H., Guiney, E. J. and Kinmonth, J. B.—(1963) *Brit. med. J.*, i, 1181.

Gould, R. J. and Schaffer, B.—(1962) *Surg. Gynec. Obstet.*, **114**, 683.

Greening, R. R. and Wallace, S.—(1963) *Radiol. clin. N. Amer.*, **1**, 157.

Hautefeuille, P. and Perrotin, J.—(1963) *Ann. Chir.*, **17**, 643.

Herman, P. G., Benninghoff, D. L., Nelson, J. H. and Mellins, H. Z.—(1963) *Radiology*, **80**, 182.

HRESHCHYSHYN, M. M. AND SHEEHAN, F. R.—(1960) *Proc. Amer. Ass. Cancer Res.*, (Abstr.), **3**, 121.
HUDACK, S. S. AND MCMASTER, P. D.—(1933) *J. exp. Med.*, **57**, 751.
JACKSON, L., WALLACE, S., SCHAFFER, B., GOULD, J., KRAMER, S. AND WEISS, A. J.—(1961) *Ann. intern. Med.*, **54**, 870.
JACOBSSON, S. AND JOHANSSON, S.—(1959) *Acta radiol.*, **51**, 321.
KENDALL, B. E., ARTHUR, J. F. AND PATEY, D. H.—(1963) *Cancer*, **16**, 1233.
KINMONTH, J. B.—(1952) *Clin. Sci.*, **11**, 13.—(1954) *Ann. roy. Coll. Surg. Engl.*, **15**, 300.
*Iidem* AND TAYLOR, G. W.—(1954) *Ann. Surg.*, **139**, 129.
*Iidem*, TRACY, G. D. AND MARSH, J. D.—(1957) *Brit. J. Surg.*, **45**, 1.
LAINE, J. B., TODD, R. S. AND HOWARD, J. M.—(1963) *Ibid.*, **50**, 866.
LEWIS, R. J. AND BEAL, J. M.—(1963) *Surg. Forum*, **14**, 112.
NORMAN, A. AND WILDER, J. R.—(1963) *J. Amer. med. Ass.*, **186**, 269.
NGU, V. A. AND KONSTAM, P.—(1964) *Brit. J. Surg.*, **51**, 101.
PEREZ-TAMAYO, R., THORNBURY, J. R. AND ATKINSON, R. J.—(1963) *Amer. J. Roentgenol.* **90**, 1078.
PICKREN, J. W.—(1956) *Roswell Park Bull.*, **1**, 79.
SHANBROM, E. AND ZHEUTLIN, N.—(1959) *Arch. intern. Med.*, **104**, 589.
SHEEHAN, R., HRESHCHYSHYN, M., LIN, R. K. AND LESSMAN, F. B.—(1961) *Radiology*, **76**, 47.
TJERNBERG, B.—(1962) *Acta radiol.*, Suppl., 214.
TURNER-WARWICK, R. T.—(1959) *Ann. roy. Coll. Surg. Engl.*, **24**, 101.
WALLACE, S., JACKSON, L. AND GREENING, R. R.—(1962) *Amer. J. Roentgenol.*, **88**, 97.
*Idem*, JACKSON, L., SCHAFFER, B., GOULD, J., GREENING, R. R., WEISS, A. AND KRAMER, S.—(1961) *Radiology*, **76**, 179.
WHITE, W. F.—(1964) *Brit. J. clin. Pract.*, **18**, 359.